# Real-world stress and control: integrating ambulatory physiological and ecological momentary assessment technologies to explain daily wellbeing

**DOI:** 10.3389/fpsyg.2025.1438422

**Published:** 2025-02-18

**Authors:** Monika Lohani, Samuel Dutton, Zac E. Imel, Patrick L. Hill

**Affiliations:** ^1^Applied Cognitive Regulation Lab, Department of Psychology, University of Utah, Salt Lake City, UT, United States; ^2^Department of Educational Psychology, University of Utah, Salt Lake City, UT, United States; ^3^Purpose Aging Transitions and Health Lab, Department of Psychological & Brain Sciences, Washington University, St. Louis, MO, United States

**Keywords:** stress, perceived control, wellbeing, real-world assessment, ecological momentary assessment, ambulatory psychophysiology

## Abstract

The current study sought to advance our understanding of the connections between stress, perceived control, affect, and physiology in daily life. To achieve this goal, we integrated hourly ambulatory physiological and experiential data from young adult participants who experienced work or academic stressors over the course of a day. Participants wore a cardiovascular monitor that recorded heart rate data continuously for 8 h while hourly random Ecological Momentary Assessment (EMA) data were collected in personally relevant settings via mobile phones to learn about stress, perceived control, and affect. The current findings provide a critical advance by demonstrating clear evidence for moderation by perceived control, wherein affective wellbeing was strongly associated with heart rate when one experienced a stressor outside their control. The innovative approach utilized in the current study in real-world settings provides further support for the value of integrating individuals' self-report and physiological experiences (e.g., the role of perceived control), as the information gained can provide critical insights into stress physiology (e.g., heart rate) and wellbeing (e.g., negative affect) connections. The present study thus provides a critical advance to the literature by connecting the literature on daily affect, perceived control, and physiological data streams. This innovation is particularly noteworthy given the general paucity of work that employs ambulatory assessments of physiological responses to daily life.

## 1 Introduction

Measurement and interpretation of affective responses associated with personally relevant stressors outside a typical laboratory setting require methodological innovation (Hoemann et al., 2021). The utilized methods must be sensitive to changes in relevant psychological domains and allow for near-real-time data collection. Physiological methods are a prime candidate for real-world data collection because they are sensitive to changes without disrupting psychological processes (Lohani et al., 2019). Similarly, Ecological Momentary Assessment (EMA) is another method that can be successfully gathered to provide context-sensitive details regarding naturally occurring daily stressors (e.g., negative work and academic concerns) to inform changes in physiological measures. In the present study of students and young adults, we investigated the feasibility of assessing naturally occurring and personally relevant affective experiences by adopting a combination of heart rate and contextualized EMA data collection.

### 1.1 Need for technology-based assessment of real-world stressors

When considering the context of daily life, work and academic stressors are some of the most common stressors experienced by students and young adults (Lohani et al., 2022). To fully capture these contexts, it is critical to integrate multiple, real-time data sources for at least three reasons. First, students and young adults differ in their interpretation of events as being stressful; even if researchers scheduled assessments to occur immediately following an exam or similar work stressor, not all individuals would see that stressful. Second, assessing individuals only at the end of the day can lead to biased responses if students fail to fully remember what occurred that day. Alternatively, individuals may have changed their interpretation of the event after having time to reappraise the situation. Third, drawbacks occur when using an end-of-day or end-of-week assessment (Lohani et al., 2023), insofar that individuals have already employed coping strategies to deal with the stressor, including calling upon their support mechanisms or distracting themselves. This last point is particularly noteworthy in the context of a student and young adult sample, given the hectic and multicomponent nature of both university life and individuals seeking new or continued employment. During the gap between stressor occurrence and later assessment, students and young adults are likely to experience multiple social interactions, other classes, and multiple environments. All of these can impact the physiological and emotional reaction to the initial stressor.

The adoption of EMA has helped overcome the methodological limitations described above by making the assessment of subjective experiences more interactive and accessible via mobile phones (Lohani et al., 2022). The value of capturing individuals' perceptions of their daily life through EMA and related methods has been underscored by decades of research linking daily stress to affective wellbeing (see Almeida, 2005; Ong and Leger, 2022 for reviews). This literature has demonstrated that individuals' affective reactivity to daily stressors can predict their health and wellbeing outcomes years into the future (Leger et al., 2018; Chiang et al., 2018; Mroczek et al., 2015). That said, this literature also demonstrates that multiple factors may moderate how stress influences the individual's wellbeing.

Critical to the current study, consistent evidence shows that perceived control may moderate associations insofar that stress holds a less deleterious effect on individuals when they perceive greater control over their lives (Cerino et al., 2024) or the stressor itself (Bhanji et al., 2016). However, only recently have researchers turned to considering perceived control in daily life. Several of these studies have focused on daily control within romantic and social relationships (Drewelies et al., 2018; Ryon and Gleason, 2018). Unfortunately, this work has typically failed to investigate daily control and affect within the same study as physiological measures were taken, which inspired the goals of the current work.

### 1.2 Importance of integrating ambulatory physiological and contextualized experiential data

Given methodological innovation, physiological indicators can now be captured in real-time during participants' daily lives, providing insights into their reactivity to current events. For instance, heart rate (Berntson et al., 2007) measures the number of heartbeats in a minute (the unit is in beats per minute or BPM). In lab-based research, it is a well-documented metric of physiological arousal and is suggested to be an excellent physiological measure in real-world settings (Lohani et al., 2019). The benefits of assessing heart rate include both the fact that this signal is strong, and it can be recorded easily in noisy environments. With reliable equipment, continuous data can be collected in naturalistic settings. Given our interest in daily stressors, heart rate was adopted as our physiological measure because it is non-disruptive to daily activities (Lohani et al., 2019).

Most physiological data to date is still collected in well-controlled lab environments; however, with technological innovation, ambulatory physiological data can be collected in real-world settings (Maselli et al., 2023). While data collection is becoming more feasible outside lab settings, just passive physiological signals are not enough to derive meaningful interpretations of psychological processes. Thus, multi-modal data (such as physiological and experiential data) must be time-synced carefully to help make inferences about psychological processes, such as daily wellbeing. The current study addresses calls from several interdisciplinary researchers to adopt innovative integration of subjective and physiological methods to understand wellbeing in naturalistic settings (Cisek and Green, 2024; Lohani et al., 2019; Maselli et al., 2023; Stangl et al., 2023).

### 1.3 The current study

The current study merged continuous physiological and subjective information over a day to understand how they interact and predict daily wellbeing. First, the current study captured daily wellbeing by assessing experiences of negative affect in everyday life (Lucas et al., 1996). This approach has been widely used with the EMA approach to gather reliable and frequent assessments of daily wellbeing (Lohani et al., 2022). In addition, we assessed participants' subjective experience with respect to their perceived control over their experience of stressors. This was intended to integrate how perceived control may modify physiological responses to naturally occurring and personally relevant stressors, thereby impacting everyday wellbeing.

Second, as a physiological measure of arousal, heart rate activity was continuously recorded and synched with subjective experiences of stress and control over personally relevant stressors in naturalistic settings. Given that affective experiences can change within an hour (Verduyn et al., 2009), we examined the relationship between subjective and physiological data streams, which were averaged hourly. Thus, we combined subjective experiences (recorded by EMA measured via mobile phone) and objective experiences (ambulatory cardiovascular activity assessment). In so doing, the present study provides a critical advance to the literature by connecting the literature on daily affect, perceived control, and physiological data streams. This innovation is particularly noteworthy given the general paucity of work that employs ambulatory assessments of physiological responses to daily life.

Thus, with the above goals in mind, we targeted the work and/or academic stressors experienced by students and young adults in their personal lives. In response to these stressors, we were specifically interested in how hourly heart rate and stress (independently or together) might explain wellbeing, as measured by negative affect. We predicted that higher hourly heart rate and experienced stress would predict higher hourly negative affect. Furthermore, we were interested in how heart rate and perceived control over work or academic stressors would explain daily wellbeing. We predicted that higher heart rate physiological response and perceived control would interact to predict hourly negative affect.

## 2 Method

### 2.1 Participants

Twenty six community members completed this 8-h-long study. They were recruited by posting ads on local bulletin boards around the city. Participants had a mean age of 27.72 years (SD = 8.71), and 84.62% were female, 1.69% were Hispanic, 88% were Caucasian, 3.85% were Black, and 7.69% were Asian. In terms of education, 7.69% reported having a GED, 15.38% reported completing high school, 3.8% were college juniors, 7.69% were seniors, 19.23% had bachelor's, 38.46% had master's, 3.85% had doctoral degrees, and 3.8% reported other. Participants were given $50 as compensation for their time. Due to technical issues or excessive artifacts that could not be meaningfully processed, data from four participants were excluded from analysis, resulting in a final sample of 22 participants.

### 2.2 Ecological momentary assessment protocol and measures

To collect EMA data, we adopted the approach we had recently implemented (Lohani et al., 2022), where participants were sent text messages and reminders via their own cell phones. SurveySignal (Hofmann and Patel, 2015) is a web-based application that allows the researcher to specify when these text messages were sent with a survey link for participants to complete. The messages were sent using a semi-random beep design: the EMA events were randomized within each hour (there were 8 h total). This way, we could connect the affective experience each hour to the physiological data each hour (using synchronized time stamps across both measures). After 15 min of lack of response, participants were sent a reminder text message to learn about their affective experiences. Also, the application was set up such that there was a minimum of 15 min between two EMA events to allow for variability in experiences. When participant clicked on the survey link sent to them via text, they would be asked the following questions about their affect.

#### 2.2.1 Negative affect scale

During each EMA event, participants reported how negative they felt in the past hour, adopting the negative affect scale (Watson et al., 1988). The following negative words were rated by participants on a 5-point Likert scale (not at all—a great deal): sadness, irritable, bored, anger, lonely, helpless, hopeless, and useless. A sum of the ratings for all words was used as the outcome variable.

#### 2.2.2 Work-specific stress and control

Also, during each EMA event, participants were asked if, in the past hour (since they were last assessed), they had experienced any work or academic stressors adapted from past work on daily stressors (Almeida et al., 2002). If yes, they were asked about the level of perceived stress they had experienced since the previous survey. Similarly, participants rated their perceived level of control over those stressful experiences. All ratings were on a 5-point Likert scale (*not at all*—*a great deal*).

#### 2.2.3 Cardiovascular activity

A research-grade ambulatory data acquisition equipment (SmartCenter; Biopac System Inc., U.S.) was utilized to collect continuous heart rate data. It collects reliable and valid cardiovascular activity. The sampling rate for heart rate data was 2,000 Hz. The utilized equipment provided fined-grained research quality information for our primary construct of interest. Post-processing continuous raw electrocardiogram (ECG) data (described in Section 2.4), heart rate (in BPM) was calculated to measure hour-to-hour average cardiovascular reactivity. A higher heart rate is linked with higher cognitive demand and workload (Berntson et al., 2007).

### 2.3 Procedure

**Figure 1:**
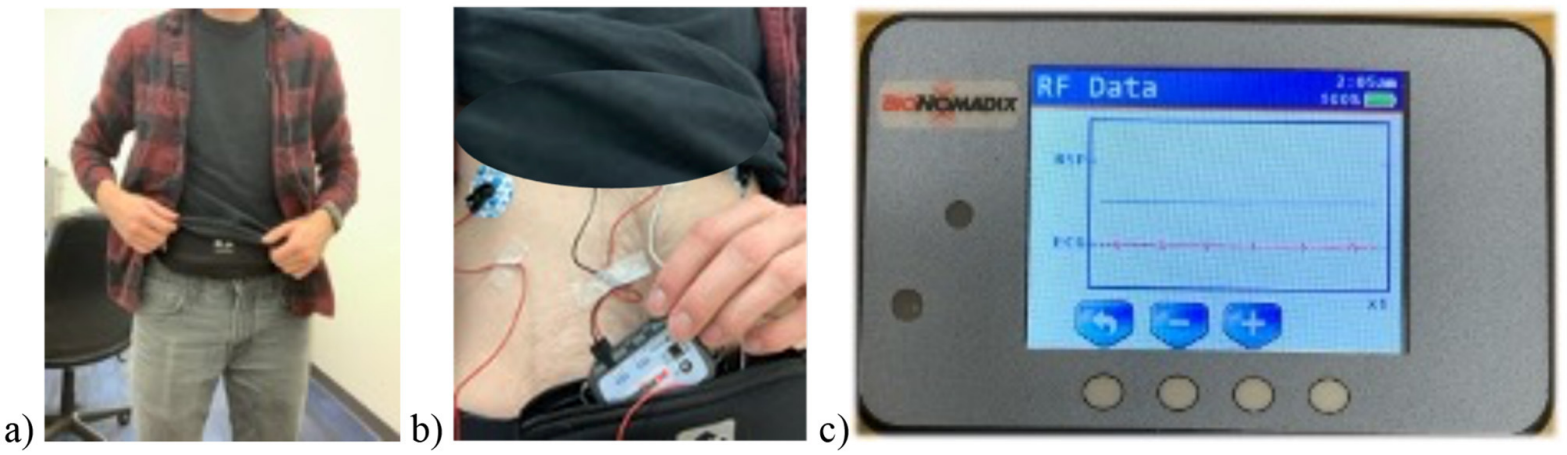
Ambulatory physiological equipment and assessment setup. **(A)** Participant wearing ambulatory equipment. **(B)** The ambulatory equipment with sensors and a small receiver placed in a running belt around their waist. **(C)** The received cardiovascular data was continuously collected for the entire duration (~8 h) in naturalistic settings.

Participants visited the lab the morning of the study, where they were outfitted with heart rate equipment. This includes cleaning the site and placing three disposable electrodes were placed at the end of the right and left ribcage and on the right collar bone (i.e., Lead II configuration; Berntson et al., 2007). After making sure the signals were being recorded as expected, the equipment was placed in a bag that the participant wore around their waist for the entire duration of the study (as shown in Figures 1A–C). Participants were informed to wear loose-fitting clothing so that the clothing would cover the equipment in the bag and was not noticeable to others. This was to ensure participants felt comfortable wearing the equipment for the upcoming 8 h.

After the physiological setup was complete, the participant's phone was configured to receive text messages over the course of the day. The participant's phone was registered to receive these messages. Finally, they were instructed not to undergo excessive physical activity but to otherwise go about their day as usual. Participants were free to go about their day (e.g., attending classes or working). They were provided with a phone number to contact if they needed any support or had questions.

All participants went about their day as they would on a regular weekday. They attended classes, worked at their jobs, and did the everyday tasks in their academic and/or work setting. Surveys were sent to participants eight times throughout the day, each hour between 11 A.M. and 7 P.M., using a semi-random beep design (Lohani et al., 2022). To better understand the immediate daily context, participants were asked if they had experienced work-specific stressors (e.g., deadlines, challenging tasks, setbacks, workload, mistakes) since the last survey. If so, they responded to questions about their perceived stress and control over that stressor. In case participants missed completing the previous EMA assessment or it was their first assessment of the day, they were asked to use the past hour-based experiences to report their responses. They also responded to questions regarding experiences of negative affect. All participants wore the physiology equipment for the entire duration of 8 h outside the lab. Once done, they removed the sticker-like ECG sensors and returned it to the researcher the next day.

### 2.4 Processing ambulatory ECG collected in naturalistic settings

**Figure 2:**
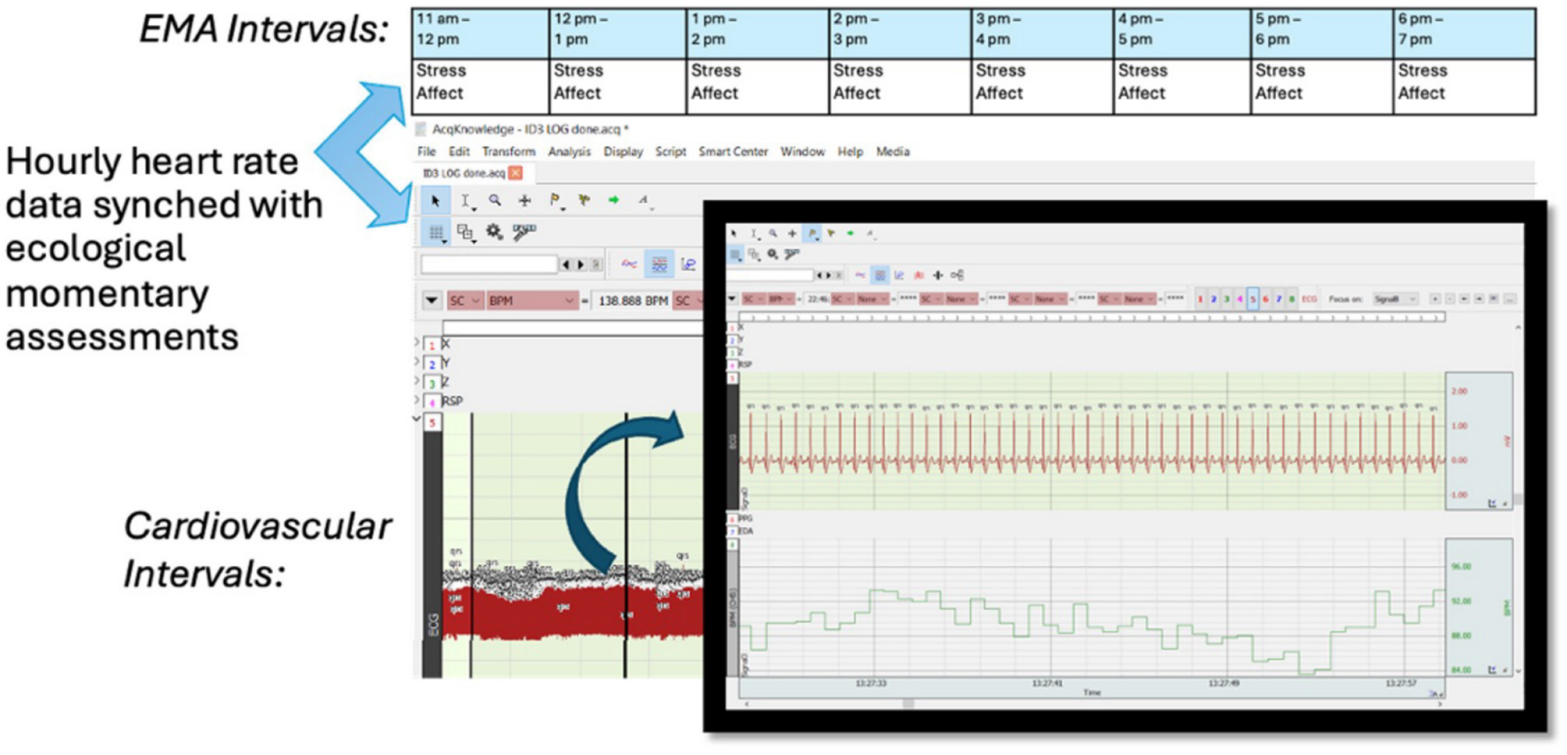
Ambulatory physiology and EMA data collected for each hour were synced together after data collection.

Post-data collection recommended processing procedures for ECG data were adopted (e.g., Berntson et al., 1997, 2007; Malik, 1996; Peltola, 2012; Shaffer et al., 2014; Laborde et al., 2017). The raw data were band-pass filtered (low-pass cutoff at 35 Hz and high-pass cutoff at 1 Hz) using a Hamming window. Next, an automatic R-wave peak detection software (AcqKnowledge software; Biopac System Inc., U.S.) was utilized to detect probable heartbeats in the raw ECG signal. This software also marked physiologically unlikely heart periods to flag potential artifacts and noise in these data due to body movements (e.g., sneezing). Next, all the heart periods were visually examined for accurate detection and then processed to ensure that physiologically improbable values were manually corrected if the software did not correctly detect them (following guidelines for ECG data; e.g., Berntson et al., 2007). Heart rate per minute was then extracted from these processed data. The heart rate was averaged across each EMA interval for each participant. Change scores were computed by subtracting participants' average heart rate of the day from their hourly heart rate. Thus, both cardiovascular and EMA data were averaged at an hourly level and synched together (see Figure 2).

### 2.5 Analysis plan

An examination of the spread of the outcome variable (negative affect) revealed it had a non-normal distribution; instead, a Poisson distribution was a better fit. Thus, Generalized Linear Mixed Models were run with maximum likelihood (Laplace approximation) estimation to account for the non-normal data. The study was particularly interested in the fixed effect of heart rate, perceived stress, and perceived control on negative affect, and specific models were run to understand their effects. They all were person-mean centered. In addition, the fixed effect of the EMA interval was included to control for time in all the models. The EMA time intervals and participant IDs were included as random effects in these models. In case the inclusion of the EMA time interval led to overfitting, then we indicated that it was removed, leaving participants as the random effects in the model. Specific models are described below. The alpha was set at 0.05 for all analyses. The original estimates from such a Generalized Linear Mixed Effects Model with a Poisson distribution generate log-transformed coefficients for each predictor in the model. Following common practice, the results in Table 1 are presented after exponentiating the coefficients to make the results easily interpretable. The confidence intervals (CI) are reported at 95%. R Statistical Software (R Core Team, 2021) was used to analyze all data.

**Table 1:** The results of the four models run to explain the outcome negative affect.

**Predictors**	**Model 1**				**Model 2**				**Model 3**				**Model 4**			
	***expB(SE)***	***CI***	***z***	***p***	***expB(SE)***	***CI***	***z***	***p***	***expB(SE)***	***CI***	***z***	***p***	***expB(SE)***	***CI***	***z***	***p***
(Intercept)	1.92 (0.48)	1.18–3.13	2.62	0.01	2.29 (0.59)	1.39–3.79	3.24	0.001	2.46 (0.64)	1.47–4.10	3.44	0.001	2.15 (0.56)	1.29–3.60	2.93	0.003
Heart rate	1.01 (0.01)	1.003–1.02	2.40	0.02					1.00 (0.01)	0.98–1.02	0.02	0.98	1.01 (0.01)	1.00–1.03	1.87	0.06
Interval	1.04 (0.04)	0.97–1.11	1.12	0.26	0.98 (0.05)	0.89–1.09	0.30	0.76	0.96 (0.05)	0.86–1.07	0.67	0.50	1.02 (0.05)	0.92–1.12	0.36	0.72
Stress					2.25 (0.26)	1.80–2.82	7.06	< 0.001	2.20 (0.26)	1.74–2.79	6.62	< 0.001				
Heart rate × Stress									1.01 (0.01)	0.99–1.02	0.93	0.35				
Control													0.87 (0.11)	0.68–1.11	1.09	0.28
Heart rate × Control													1.03 (0.01)	1.01–1.04	3.88	< 0.001
**Random effects**																
σ^2^	0.25				0.27				0.27				0.27			
τ_00_	0.90 _ID_				0.32_ID_				0.32_ID_				0.39_ID_			
τ_11_	0.01 _ID.Interval_															
ρ_01_	−0.52 _ID_															
ICC	0.74				0.55				0.55				0.59			
Marginal *R*^2^/Conditional *R*^2^	0.02/0.74				0.37/0.72				0.37/0.71				0.14/0.65			

## 3 Results

### 3.1 Compliance and descriptive information

Participant compliance was 87%, with all participants responding to over half of the hourly assessments: eight participants responded all eight times, seven responded seven times, five responded six times, and two responded five times. Negative affect had a mean of 3.08 (SD = 4.26). Heart rate had a mean of 88.41 beats per minute (SD = 13.23), which aligns with the normal heart rate expected in young adults. Work/Academic stress level had a mean of 1.36 (SD = 1.01), and perceived control had a mean of 1.96 (SD = 1.2).

A few examples of work and academic stressors were reported by participants (which were optional): disagreements with coworkers and the project not working out properly, issues finishing work-related tasks, finding mistakes in work done, stress about exams, and difficulty meeting deadlines. In response to these personally relevant stressors, our focus was on the experience of stress and their perceived control and wellbeing, which we monitored over the day using EMA and connected to physiological responses.

### 3.2 Heart rate and perceived stress rating separately predicted negative affect

The Model 1 included a person-mean centered heart rate to explain negative affect. The fixed effect of heart rate was a significant predictor of negative affect, [*exp*β(*SE*) = 1.01(0.01), *z* = 2.40, *p* ≤ 0.02, 95% CI = (1.003, 1.02)]. An increase in heart rate was linked to an increase in negative affect. See Table 1 for additional details on the fixed and random effects. Similarly, Model 2 was run to examine the fixed effect of person-mean centered perceived work/academic stress on negative affect. The inclusion of time interval as a random effect led to overfitting; hence, it was removed with only the participant as the random variable. An increase in perceived stress predicted increased negative affect, [*exp*β(*SE*)= 2.25(0.26), *z* = 7.06, *p* < 0.001, 95% CI = (1.80, 2.82)]. Additional details are reported in Table 1. However, when both the fixed effects of heart rate and perceived stress were included together in Model 3, their interaction was not significant (*p* = 0.35). Thus, higher levels of negative affect were separately associated with higher heart rates and perceived stress, but the two predictors did not interact to explain negative affect.

### 3.3 Heart rate and perceived control rating separately predicted negative affect

A final model was fit to explain negative affect based on heart rate and perceived control of work/academic stressors. Model 4 included a person-mean centered heart rate, person-mean centered perceived control, and their interaction as fixed effects and participant and time interval as random effects. However, the inclusion of time as a random effect led to overfitting. It was removed from Model 4, leaving the participants as the random variable. Negative affect was predicted by the interaction term between heart rate and perceived control [*exp*β*(SE)*=1.03(0.01), *z* = 3.88, *p* < 0.001, 95% CI = (1.01, 1.04)]. Negative affect was not separately predicted by the fixed effects of heart rate (*p* = 0.06) or perceived control (*p* = 0.28). See Model 4 in Table 1 for details on random effects.

**Figure 3:**
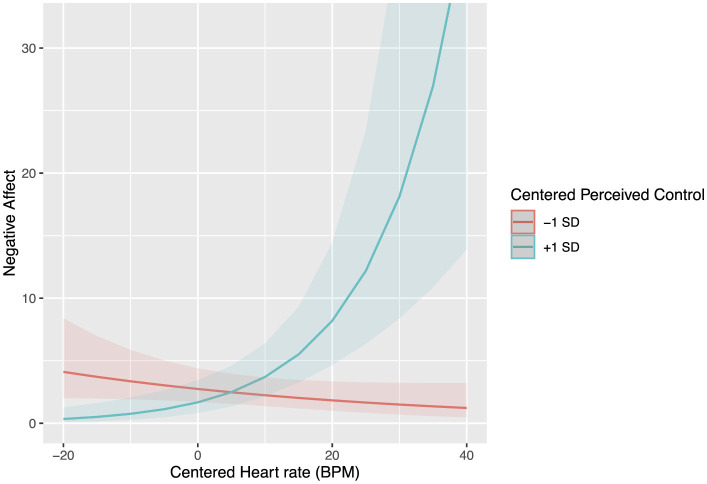
(Person-mean centered) Heart rate in BPM and perceived control interaction predicted negative affect.

In order to interpret the interaction term, the link between the outcome and heart rate was plotted at high (+1 SD) and low (−1 SD) levels of perceived control (see Figure 3). A simple slope test revealed that a high level (+1 SD) of perceived control heart rate was associated with higher levels of negative affect [β*(SE)* = 0.04(0.01), *z* = 3.99, *p* < 0.001]. However, at low levels (−1 SD) of perceived control, there was no effect of heart rate on negative affect [β(*SE*) = −0.01(0.01), *z* = 0.85, *p* = 0.40). Therefore, the positive relationship between heart rate and negative affect was present only at high levels (+1 SD) of perceived control.

## 4 Discussion

### 4.1 Benefits of a multimethod approach and innovation

The current work is among the initial studies to combine ambulatory cardiovascular data and EMA in real-world settings to gain an understanding of work and academic specific perceptions and their potential influence on affective wellbeing. The results show that real-world affective experiences in response to personally relevant work and academic stressors can be explained by combining information from the subjective experiences of the responder and objective cardiovascular activity. In particular, subjective perceptions of control over stressors and heart rate changes together explained daily wellbeing. Furthermore, heart rate was linked to daily wellbeing at an hourly level. These findings align with recent calls for implementing a multi-modal approach to understanding cognition in everyday life (Cisek and Green, 2024; Lohani et al., 2019; Maselli et al., 2023; Stangl et al., 2023).

The current findings advance our understanding of the connections between affect and physiology in daily life. Previous work has demonstrated that self-reports of affective wellbeing are often associated with physiological arousal, but these associations are typically weak in magnitude and may only be present at the within-person level (Schwerdtfeger and Gerteis, 2014; Zawadzki et al., 2017). However, most of this EMA work has focused on naturally occurring fluctuations without integration of work-related stressors or perceived control over these stressors. The current findings thus provide a critical advance by demonstrating clear evidence for moderation by perceived control, wherein affective wellbeing was strongly associated with heart rate when one experienced a stressor outside of their control. The innovative approach utilized in the current study in real-world settings provides further support for the value of integrating individuals' self-report and physiological experiences (e.g., the role of perceived control), as the information gained can provide critical insights into stress physiology (e.g., heart rate) and wellbeing (e.g., negative affect) connections.

The present study focused on perceived daily stressor control in order to better contextualize the study within the daily experience of young adults. However, work comparing general to specific control beliefs would provide valuable insights into which control aspects are most critical to intervene upon, particularly given that these constructs hold differential trajectories across adulthood (Cerino et al., 2024). Indeed, a critical advance of this work is the focus on the relevant stressors for the given developmental period, including academic stressors for students and workplace stressors for young adults. This work heeds calls for understanding how best to promote student wellbeing, in the face of a growing mental health crisis among young adults (Tan et al., 2023). Understanding how best to help students reduce anxiety and stress has become a critical need, particularly among the current generation of students who have dealt with the COVID-19 pandemic. Research shows that university students were vulnerable to greater disruption during the early weeks of the pandemic with respect to their ability to carry out their personal purposes and life aims (Hill et al., 2022). The after-effects of this disruption to purposeful engagement likely continue to this day, elucidating the need for the present research into how students and other young adults are reacting to stressors in daily life. Future work should consider whether the current cohort differs from those yet to come who did not face the environmental challenges of COVID-19 during their formative years of adulthood.

### 4.2 Limitations and future directions

Some limitations of this study need consideration as directions for future research. First, our sample was limited and was disproportionately female, which limits the ecological validity. With a larger and more representative and diverse sample, researchers could incorporate individual differences to explore moderation by state- and trait-level perceived control. Future work with a larger sample size would also allow for modeling naturalistic behavior, individual difference analysis, and benefit from machine learning approaches (Azari et al., 2020; Maselli et al., 2023). Second, the current study adopted a randomized-beep design where a random sampling of experiences was conducted. However, providing participants a way to manually report significant stressors or specific events would provide additional details, and future work should incorporate this option. Third, knowing more specific details about the duration and relevance of work and academic stressors may also help better understand the physiological markers of stressful events. Finally, the study occurred over the course of a day; however, a lot more could be understood by expanding to a longer period of longitudinal data collection (e.g., days and weeks).

### 4.3 Conclusion

Affective wellbeing to personally relevant stressors was explained by a combination of perceived stressor control and cardiovascular activity. Together, this work highlights the importance of multi-modal assessment and integration to understand the connections between everyday stressors and physiology in daily life. Extending lab-based work, the study also demonstrates the feasibility of collecting ecologically valid and personally relevant multi-modal data. The findings provide empirical evidence supporting the importance of incorporating contextual and ecologically valid methods that capture experiential and physiological responses to naturally occurring everyday stressors.

## Data Availability

The raw data supporting the conclusions of this article will be made available by the authors, without undue reservation.
